# Engaging faith communities in public health messaging in response to COVID-19: Lessons learnt from the pandemic in Ituri, Democratic Republic of Congo

**DOI:** 10.3389/fpubh.2022.916062

**Published:** 2022-09-13

**Authors:** Amuda Baba, Liz Grant, Nigel Pearson, Emma Wild-Wood, Jean-Benoit Falisse, Yossa Way, Sadiki Kangamina

**Affiliations:** ^1^Universite Anglicane du Congo, Faculty of Theology, Bunia, Democratic Republic of Congo; ^2^Institut Supérieur de Techniques Médicales de Bunia (ISTM-Bunia), Bunia, Democratic Republic of Congo; ^3^Usher Institute, University of Edinburgh, Edinburgh, United Kingdom; ^4^Independent Public Health Consultant, Bunia, Democratic Republic of Congo; ^5^Faculty of Divinity, University of Edinburgh, Edinburgh, United Kingdom; ^6^Centre of African Studies, University of Edinburgh, Edinburgh, United Kingdom

**Keywords:** faith communities, public health messages, Democratic Republic of Congo, COVID-19, health promotion

## Abstract

**Purpose:**

To understand challenges faced by faith leaders in the Democratic Republic of Congo (DRC) in engaging with current public health strategies for the COVID-19 pandemic; to explain why long-standing collaborations between government, faith-based health services and leaders of faith communities had little impact; to identify novel approaches to develop effective messaging that resonates with local communities.

**Methods:**

A qualitative participatory research design, using a workshop methodology was deployed to seek opinions of an invited group of faith leaders in the DRC provinces of Ituri and Nord-Kivu. A topic guide was developed from data gathered in prior qualitative interviews of faith leaders and members. Topics were addressed at a small workshop discussion. Emerging themes were identified.

**Findings:**

Local faith leaders described how misinterpretation and misinformation about COVID-19 and public health measures led to public confusion. Leaders described a lack of capacity to do what was being asked by government authorities with COVID-19 measures. Leaders' knowledge of faith communities' concerns was not sought. Leaders regretted having no training to formulate health messages. Faith leaders wanted to co-create public health messages with health officials for more effective health messaging.

**Conclusion:**

Public trust in faith leaders is crucial in health emergencies. The initial request by government authorities for faith leaders to deliver set health messages rather than co-develop and design messages appropriate for their congregations resulted in faith communities not understanding health messages. Delivering public health messages using language familiar to faith communities could help to ensure more effective public health communication and counter misinformation.

## Introduction

During the COVID-19 pandemic, misinformation about the disease circulated widely in every country. The Director General of the World Health Organization noted that the world, was “not just fighting an epidemic, we are fighting an infodemic” of false information about COVID-19. This fight was, as for other diseases, a matter of debunking myths and circulating the latest and most accurate information validated by health authorities.

Some literature has begun to unpack the dynamics during the COVID 19 pandemic (1, 2). It has focused either on key demographics known to be suspicious of health authorities or on low- and middle-income contexts where trust in health authorities is low. Little has been written on contexts of widespread violence and conflict where trust in authorities has almost totally broken down. Tar Gay (3) examines the reactions of religious leaders to COVID-19 in Myanmar during military takeover but does not engage with public health questions.

This study examines the case of Ituri in eastern Democratic Republic of Congo (DRC) where the engagement of faith communities in public health messaging came against the background of decades of the State's failure to provide essential services and basic security. Discovering the public health situation during COVID-19 in an area of extreme political fragility is an important element of this research. Our research sought to 1/ understand how faith leaders (in a situation where trust in state authorities is low and risks—disease and security-related—are high) perceived the public health response to COVID-19 and 2/ curate their recommendations for responding to future health emergencies. We show that local faith leaders were aware of the effects of misinformation and public confusion about COVID-19 but often felt powerless to respond effectively. Faith leaders were rarely treated as equal stakeholders in health emergencies. The approach of providing health messages for faith leaders *simply* to transmit to their communities was not effective.

By working together to design and develop public health messages, public health practitioners and faith communities could reach a wider public more effectively. Public health practitioners could learn from faith leaders ways to best recognize and respond to the complex social and spiritual environment which shapes health understanding and health seeking behavior.

Before introducing the research and findings we present a rationale for research and explain the particular contextual elements to health-care in DRC, and COVID-19 and insecurity in Ituri province.

### Rationale for the research

The Lancet Series on Faith Based Health Care examining the faith-public health nexus acknowledged the role of faith at all levels in public life—individual, community, national and international (4), even while “Faith factors are not always taken into account in public health discourses,” (5). Faith communities are among civil society organizations that have “capacities to bridge and build trust” in insecure situations (6). They are responsible for between 30 and 70% of healthcare provision in Africa (7), often filling gaps in coverage, providing preventative, curative, rehabilitative and palliative care services, and promoting public health messages. In 2021 in response to the pandemic, the World Health Organization (WHO) published their “Strategy for engaging religious leaders, faith-based organizations and faith communities in health emergencies.” In it they identified that the COVID-19 pandemic required greater collaboration, “a holistic and integrated response across society, religious leaders, faith-based organizations, and faith communities.” Seeking greater collaboration developed, in part, from a recognition that the pandemic tested people's trust in public health messaging and those behind them (8, 9). The question of trust is not new in public health; among others, the seminal work of Gilson (10) has established that public trust is central to the legitimacy of health systems and public health interventions in general (11). In contexts where trust in authorities, health related or not, is low, a common strategy has often been to forge alliances with those who are seen as more legitimate. Often first among them are religious leaders, and there are numerous examples of public health strategies mobilizing them to reach key demographics (such as elderly with diabetes in Mexico: Rivera-Hernandez (12) and tackle specific conditions [e.g., hemoglobinopathy; (13)].

The Faith and COVID-19 Resource Repository was launched by the Berkley Center for Religions, Peace and World Affairs at Georgetown University, the World Faiths Development Dialogue and the Joint Learning Initiative to curate the evolving contribution and critique of faith communities. Its review of religious responses to COVID-19 identified a wide and diverse series of interventions from faith communities—often understudied actors because much literature focused on faith-based medical or development organizations. While faith communities have a unique place—with a physical reach into almost every community, alongside a powerful social, and spiritual connective capacity, their role globally has not translated fully into a complementarity of effort that is needed for rapid pandemic responses. The reasons are complex, but an important starting place for future collaboration with faith communities in pandemic preparedness is a better understanding of relationships between the public health sector and faith. Cognizant of the dangers of taking an instrumental approach to faith communities our research sought to understand “the cultural and faith-based concerns of faith communities related to health,” [(14), p. 4] so “that community and faith-led approaches are championed” [(14), p. 2]. To do this we examined the views of local faith leaders on the role they play in health and wellbeing, and how their engagement might best be supported.

This article is part of a larger humanities research project designed to enable the conversations between Theology and Religious Studies and Global Public Health discourses to develop a social understanding of disease (15). Two prior articles coming to press present findings that inform this study. One sought to characterize and classify the range of perceptions of COVID-19 among members of faith communities in Nord-Kivu and Ituri (16). They include the view that COVID-19 is a natural viral disease and that COVID-19 is a supernatural disease caused by evil spirits or the devil. The range of opinions highlight considerable confusion about the disease and measures taken in reaction to it. Additional research (17) shows that relations between faith communities and especially faith leaders and government changed as a result of responses to COVID-19 epidemics, with faith communities traditionally more distant from the State and authorities (medical and more generally) becoming closer to them through compliance to public health restrictions while at the same time becoming increasingly frustrated by the public health discourses.

This paper provides an additional unique perspective exploring faith leaders understanding of complexity of health messaging and analyzing their opinions on how public health strategies and co-operation between stakeholders affected community engagement with public health messaging during COVID-19. Contrary to our other papers that analyzed contextual aspects, this paper explores how strategies and co-operation could be improved from a very practical standpoint.

#### Context

Our research took place in a particular context of health-care delivery that expected the positive engagement of faith communities in public health messaging. To counter the spread of misinformation, and to increase sensitization, health authorities in the Democratic Republic of Congo (DRC) in charge of the COVID-19 response called for the support of faith leaders to deliver public health messages to their faith communities (Vatican News, 2020), in line with WHO recommendations (No. WHO/2019-nCoV/Religious Leaders/2020.1). The strategy was deemed appropriate in a country where 99% of the population is a member of a faith community (18) and where faith-based institutions are central in the delivery of health-care for the Ministry of Health in a complex, negotiated system of co-management [(19, 20), p. 78–9]. Furthermore, in DRC faith partnerships and informal and personal relations between faith and health sectors are strong; many government officials and health workers are committed members of faith communities. However, in practice, anti-COVID-19 government measures were neither universally followed nor sustained (21). While the majority of faith communities engage with the national health plans, a small number of faith leaders “refuse modern medicine” including nationally established programmes such as the measles vaccination [(22), p. 2]. During the COVID-19 pandemic he situation varied between faith communities and locations: some, like the Catholic Church with its significant resources and close relations with national and international faith-based medical and aid organizations, were actively involved in promoting public health measures to protect against COVID-19 (23). Others much less so. Overall, there was confusion about the nature of COVID-19 and the public health mitigation measures (16, 24).

This national approach of health-delivery faced particular challenges in Ituri. North-east DRC is frequently overlooked in research because of its insecure situation. Political insecurity has affected north-east DR Congo for 26 years. More than 130 armed groups are active in the provinces of Ituri, North Kivu and South Kivu (25). They increase food insecurity, poverty and sexual violence (26). The violence escalated in Ituri from January 2020 (27) prompting the instigation of a state of emergency in May 2020 and further internal displacement into Bunia, the provincial capital of Ituri (28). The identification of the first case of COVID-19 in the DRC occurred on 10th March 2020 (29). Public health measures were broadcast for application from 19th March 2020 (30). A nation-wide state of emergency was announced on 24th March 2020, which included the closing of places of public worship until 10th August 2020, when they were permitted to open with small congregations and observing preventative measures developed by the government.^1^ The first case of Covid-19 in Ituri Province was identified on 30th March 2020, in Nyankunde, 45 km south of Bunia (31). In early April 2020, Ituri was among the 4 provinces which had registered Covid-19 cases, with Kinshasa, North Kivu and South Kivu (32). There was concentration of cases and suspected cases in Bunia, and also in Aru and Mahagi, two bordering towns of Ituri with Uganda (33, 34). The first test equipment in Ituri was operational at the referral hospital of Bunia. Even so, the official number of Covid-19 cases in Ituri, which has an estimated population of close to 4 million people, would only pass the 1,000 mark at the end of July 2021 (and with only 57 registered deaths) Radio Okapi (35, 36). The political insecurity, often appeared to pose a more immediate threat than COVID-19 and public health restrictions were met with some resistance.

## Methods

The research focuses on the city of Bunia, the capital of Ituri province. Initial research was conducted in other places in Ituri and Nord-Kivu but by the time of this focus group the security situation had deteriorated. Bunia was one of the few places in Ituri that was accessible for the research (from a security perspective) and offered a concentration of different religious denominations in the two provinces (in many cases often regional headquarters are based in the town) that could enhance the relevance of our observations. This meant that our research would be primarily urban.

### Sampling, recruitment, and participants

Drawing on a qualitative participatory research design, and using a workshop methodology (37) we held a participatory workshop of faith leaders from Bunia, with leaders from different faith communities coming together in one interactive space. The workshop technique enables opinions on specific topic to be gathered using focus group interaction and discussion (38). From the database developed during the first stage of the project we invited participating faith communities to nominate delegates who were faith leaders in their own communities in Ituri Province to participate in the workshop. Due to COVID-19 and heightened political insecurity restricted travel and social interaction, only Bunia resident faith leaders were able to participate. Eleven faith leaders took part (10 men and one woman). The gender divide reflects the imbalance in leadership of faith communities in Bunia. Of Bunia's many different faith communities, 90% are from a Christian tradition. The identity of participants has been protected by listing their involvement by general affiliation:

- Two participants (one male, one female) from the Roman Catholic Church, the largest Christian denomination in DR Congo.
- Six participants of three member communities of the Protestant association, Eglise du Christ au Congo (ECC).
- One Muslim participant. Estimates of Muslim adherents vary, but probably 5–10% of Congolese are Sunni Muslim.
- Two participants from two member communities of the Pentecostal association of Eglises du Reveil au Congo (ERC).

The Catholic Church and ECC members support medical delivery *via* their own denominational faith-based health organizations that run hospitals, clinics and health districts in Bunia and elsewhere in DR Congo. The ERC churches and the Muslim community do not run medical facilities in Ituri. Participants in the workshop held positions of authority within their own faith community and the immediate area. Each had influence in church and civic matters in Bunia and lived amongst the communities they served. They represented those who could make a local difference to the compliance with public health messages. The scope of the findings is limited by the small number of participants and the self-selection implicit in those who accepted the invitation to participate. Lam-Te-Kwaro, an indigenous faith community that recourses to ancestor veneration as part of its response to disease, participated in earlier interviews but did not attend this workshop. The small group discussion allowed faith leaders to speak at length, enhancing the quality of their contributions.

### Data collection and analysis

The research workshop was organized on 29th June 2021 and conducted in French, the language used for formal events, in which all participants were fluent. Participants signed their consent to participate and be recorded. The workshop set out the challenges of COVID-19 by presenting preliminary findings from earlier interviews. This data described the perceptions about COVID-19 among faith communities and the response to public health messaging by people in faith communities (16, 24). Semi-structured, curated questions were used to facilitate an open focus-group conversation on the following topics:

- Perceptions of COVID-19 from the data presentation.
- Perceptions of public health restrictions from the data presentation.
- Current views of government public health strategies.
- The relationship between faith and the pandemic, and whether faith texts and rituals speak into the COVID-19 situation.
- Contribution of faith leaders during epidemics to local and national health agendas.

We developed a coding framework based on the topic guide above and the themes emerging from the data (39). From a curation of the different topics mentioned above, each topic, themes and sub-themes were identified. Five main themes (identified in sub-headings below) emerged from the data, with sub themes connected to the absence of systems, supports, resources, training and trust,.

## Research findings

Faith leaders identified specific problems and suggested solutions:

### Summary

1. Faith leaders described how members of their congregations were confused and how misinterpretation, misinformation and disinformation about COVID-19 were leading to public confusion. They attributed this to a lack of engagement with public health measures
2. Faith leaders said they themselves *lacked capacity*–health knowledge, resources and time—to implement government measures.
3. Faith leaders said they *lacked engagement with authorities, who did not seek their expertise* i.e., the embedded knowledge of their communities.
4. Faith leaders see the need for, and *would like to co-create, health messages* so faith communities can better understand them.
5. Faith leaders lacked and would *welcome public health training and greater engagement with health authorities to co-create the messages* to their faith communities.

### Confusion and misunderstanding

Faith leaders described the levels of misinformation in the community about COVID-19 and their uncertainty about an appropriate response. They considered that the messaging was contradictory and unclear: “*even the people who brought us the measures, [and] told us to listen to the reality of things and we found somewhere also between them there was also confusion. Even today the confusion continues*.” Two members of the ERC association were particularly vocal on this point. One said, “*Each person understands it in their own way, that's what makes it a bit difficult*.” There was a lack of trust between health officials and faith communities which some communities felt more acutely than others: “*the structures that were supposed to give information relied more on obligations and recommendations rather than first sensitizing people to understand what it was all about*” said one, while another explained, “*secrets of scientists…who are doing research on COVID-19 are not made clear and that pushes others to think that it is the devil who is at the base*.”

### Insufficient capacity to implement government measures

The concerns from faith leaders about their own lack of ability to do what was being asked by the health sector focused on three areas: a lack of knowledge, resources and time.

Faith leaders discussed their own limited *knowledge* of the disease and described their uncertainty about appropriate responses to their communities' misunderstandings. To counter the lack of accurate information participants spoke of the need to “*gain the trust of the people in charge*.”

A lack of *resources and* inequality in resourcing as identified in terms of material resources and confidence and institutional support. Faith communities with close connections with clinical services, through partnership or direct delivery, were better able to respond to the requests from government than those who had few connections. A participant representing the most well-resourced faith community, the Roman Catholic Church, spoke of its ability to act independently when state interventions were not forthcoming, and its action alongside government initiatives where they were in place.

Some participants described how the *timing* had been counter-productive. The sudden implementation of public health measures had created great hardship for individuals and in faith communities in Bunia. A representative from an ECC community explained, “*Instead of imposing a period of confinement, perhaps we should have multiplied the opportunities for awareness, for explanation, to show the gravity of the disease to people*.” While recognizing the importance of taking rapid action, participants explained that lockdowns, instigated with little information or prior warning, were challenging to administer. They reduced the resource of faith communities further, in terms of finance and pastoral care.

Despite these difficulties, participants also identified the positive contribution of faith communities to tackling the COVID-19 pandemic. A Catholic representative said churches were involved from the beginning in “*raising awareness against this pandemic*,” and leaders risked their lives to pray with the sick. She believed that across faith communities, “*the religious denominations did their job. Even if they were not all committed to the same level, for the most part they did their job*.” Other leaders mentioned untapped resources of trusted relationships with members of faith communities that could strengthen public health measures.

### Lack of—and need for—engagement with the authorities

Despite the difficulties they identified, most faith leaders considered that faith communities had demonstrated a commitment to being “health responsive,” They thought that this commitment was often overlooked by health officials. One said, “*faith community leaders should work in collaboration with the government*” to shape the public health measures that were proposed, adding that faith leaders, “*must not look as if they put up with the decisions coming from government; but they must work in close collaboration in order to give the real message from this government*.” Authorities, one said, should recognize the close, trusted relationship they had with their communities, “*We are opinion leaders and we have people in our hands*.” This was because “*we live with the population 24 h a day and we are with them in times of misfortune and in times of happiness... They* [public authorities] *should consult us before* [a health situation] *might degenerate*.”

Participants spoke in agreement of the steps that could be taken by faith leaders in future outbreaks of disease in order to improve outcomes. One ECC participant said, “*If there is a problem that affects physical health, those who see spiritual health should also be interested in order to get things on track*.” Faith leaders spoke of the need for Christians of all denominations to help each other in this endeavor and offer practical signs of love, “*as was done in the primitive* [early] *church*.”

### Desire to co-create public health messages

Workshop participants discussed *how* they might engage with health authorities. They were in broad agreement about the importance and the value of public health messages. The Muslim participant said, “*God, before anything else, is a God of order. God loves order. So we find these measures salutary for a strategy which goes along with our belief and community*.” Co-creation of public health messages became the first solution to the challenges the participants had raised. An ERC representative said that faith leaders “*must help the public health officials to popularize the message by explaining that COVID-19 is real and it can be dangerous*.” There was general assent to the suggestion that an “oversight committee” be formed to respond to future crises. It would not impose measures but would see where the population had difficulty in compliance because of a lack of basic amenities, like water, soap or face coverings.

All leaders discussed the use of scripture and the value of leaders preaching about COVID-19 using verses from the Bible or Qur'an: “*the faithful will be convinced… even God has already talked about it*,” said the Muslim participant. There was common consent that the preventative public health measures were not in contradiction to religious teaching except where poor communication about the messages caused some members of faith communities to see them as contradictory. Explaining health measures using the language of scriptures could facilitate better comprehension: “*almost all the barrier measures are in the Bible… quarantine and cleanliness were expected of the children of Israel*.” Participants offered Leviticus 13, Exodus 30 and Isaiah 26 as examples of hygiene, disease prevention and cure. Faith leaders whose views about COVID-19 differed from a bio-medical view at the beginning of the workshop, entered fully into the discussion of which faith texts resonated with COVID-19 prevention measures.

### Desire for training to explain the messages to their faith communities

Faith leaders regretted a lack of appropriate training that could enable them to explain measures using familiar idioms. Participants wanted to understand disease transmission and prevention, medicines that can cure the disease, the role of the vaccine, and the possibility of becoming infected after being vaccinated. All participants spoke of the value of trained, knowledgeable faith leaders, “*so that people can really understand the disease and come to accept that this disease exists and see the seriousness of this disease*.” They identified training as a first step in renewed partnerships with health authorities in order better to respond to ongoing health crises.

Participants with less institutional and international support and less historic involvement in healthcare delivery were less confident of their ability to raise awareness among their communities. The ERC representatives, whose churches were not involved in state-sanctioned health delivery and who had initially expressed doubts about COVID-19 public health measures were hesitant about working with the state. By the end of the workshop they expressed a willingness to join programmes of training to understand and support public health measures, even when their views on healing and disease differed from public health norms. They entered fully into the discussion of biblical health measures that were similar to COVID-19 prevention measures. The facilitated workshop allowed dynamic conversation to emerge, with participants being able to discuss public health using familiar reference points.

The majority of local faith leaders considered themselves ill-equipped to provide good advice on COVID-19. Their communities lacked knowledge and so misinformation circulated as a result. To counteract this, leaders expressed their desire to be trained in order to understand the disease and better counteract misunderstandings. Faith leaders did not want to repeat messages they themselves did not understand. Faith leaders spoke of the importance of co-creating messages with health leaders for effective communication among faith communities. They explained how they could translate messages into a faith register using meaningful language that would be familiar to their congregations people, i.e., infused with reference to spiritual beings and regularly referring to tenets and texts of the faith community. This method could be especially valuable for faith communities who hold non-biomedical views on disease. Faith leaders also believed that they would be more able to identify disease containment and mitigation measures that would be deemed fairer and more accessible by more people, and that they could help advise on what strategies would be most appropriate for religious gatherings as well as market places, shops etc.

Faith leaders shared their perspectives on the need to exchange ideas with health practitioners. Mutual listening and learning—as seen in the participatory workshop—could help to establish trusting relations which are essential for an effective public health response. Discussing misinformation and sharing distinct views about healing could improve the understanding of the social impact of public health measures. Dialogue can open up opportunities for addressing tensions within local communities.

These findings showed that there were many shortcomings in the collaborations between public health authorities and faith leaders. They described how they wanted to “communicate health information … that aligns scientific evidence with religious values” in collaboration with national health services [(14), p. 5]. The support by participants for public health concerns was accompanied by criticism of COVID-19 public health measures.

## Practical considerations and recommendations

In places of political insecurity where health systems are overburdened and poorly resourced, like DRC, involving faith leaders effectively in a health emergency requires a level of organization that is challenging, even when much health delivery is supported by faith communities [(40), p. 176–8]. A set of recommendations were developed from the themes which the faith leaders had identified within the workshop. Whilst the research team in Bunia engage stakeholders in considering practical steps our practical recommendations remain tentative. They include:

- Formation of a group of representatives from across the religious and denominational spectrum that is ready to act in health emergencies and on-going challenges. Ecumenical and interfaith networks could be used in identifying some representatives. Marginal groups should be deliberately included. Faith communities that are not part of the “mainstream” should be intentionally “drawn into collaboration” [(40), p. 184].
- The group would have training by health officials. It would also provide health officials with better understanding “of the heterogeneity of viewpoints, both within and between faiths, and their effect on health care” (4).
- In a health crisis the group could work with health officials to apply training to construct contextually communicable health messages about disease and its control and provide the theological reflections for their communities that undergird an emergency response.
- Participation needs to be seen as prestigious or to prompt a sense of civic duty to encourage representatives from all faith communities to engage.
- Such a forum of faith leaders might meet regularly to discuss a broader remit of health challenges, disaster preparedness and response. It could act as a knowledge hub, sharing how health messages are best communicated, preparing faith materials on disease and exploring the variety of theologies of healing that exist in different faith communities. This would encourage an “increased appreciation in faith leaders of the effect of their teachings on health care” (4).

## Discussion and conclusion

The paper analyzed a workshop which discussed how public health messaging within faith communities would be made more effective if health practitioners engage local faith leaders before the outset of a health emergency.

This is the first study in DRC to explore through a workshop methodology the perspectives of faith leaders with regard to public health responses, in the Bunia region in the context of the combined crises of COVID-19 and conflict. Limitations of this study were its small numbers, and the distinctively small area in which the study took place. We sought to recruit more participants coming from the range of theological and religious perspectives representative in the region but escalating risk factors of insecurity hindered participation However while we suggest that caution is needed when generalizing these findings to other regions in Central Africa we do believe that they are substantive with rich open data collected, and coding being undertaken by experienced researchers.

The public role that faith leaders in DR Congo already play in a society of extensive faith-based health-delivery contributed to the expectation that faith leaders would transmit important government messages during the COVID-19 pandemic (41). Yet local leaders lacked knowledge, resources and training to support public health measures against COVID-19. Prominent national leaders from large established faith communities that support biomedical health delivery were more likely to be able act as equal partners in health emergencies. Local leaders, those from smaller communities and those who doubted a biomedical view were often overlooked and not regarded as stakeholders by health officials. Yet local faith leaders are key to health delivery. They support individual and communal wellbeing. They are often highly respected role models and have decision-making power and influence in their local communities. Many are willing to support measures to reduce disease. If all faith leaders are to be treated as stakeholders—that is, partners who have an equal responsibility and desire for improving the health of society—health officials should adapt their practice to the social understanding of disease presented by local faith leaders.

This paper recommends that, as key stakeholders in health delivery, local faith leaders should participate in the epidemic response co-ordination structure before the onset of a health emergency. Faith leaders can interpret the problems and devise messages that make sense to them and their constituents. This is likely to contribute to changing the initial perceptions and misinformation of the disease and may increase willingness to comply with health measures in a range of faith communities. With health officials, faith leaders could co-create a public health message focused on disease description, means of transmission and means of prevention that included sacred texts, beliefs and practices that are consonant with the health message. With faith leaders, health officials could consider how to adapt health responses and communication to the concerns of the different faith communities. A similar initiative has been proposed in Nigeria [(42), p. 115–8] arising from the problem of misinformation circulating among Christians in Abia state and in Romania (43) with the Orthodox church.

Our findings on the challenges identified by faith leaders that functioned as obstacles for creative engagement, and the recommendations from faith leaders of approaches going forward—complement the three areas identified by the Berkley Center in their examination of the role of faith communities in public health activities (44). Faith communities can contribute to global health advocacy, delivery, and policymaking. These three areas of creating and sustaining dialogue between government and religious partners, of investing in broader cross-sectoral and inter-sectoral collaboration with faith leaders, and of practical and immediate actions to vaccinate all point to the need for equity in partnership. Equity in partnership is key. Engaging leaders of faith communities at the outset of a health emergency could produce messages that are understood, followed and owned. Such messages take into account peoples' beliefs and fears, and recognize that the language of faith may express the risks, values, and importance of taking health actions. This co-production of knowledge could contribute to managing health emergencies and on-going challenges in fragile and conflict affected settings. It requires health officials to see all faith leaders as equal partners able to contribute to the social understanding of disease.

## Data availability statement

The raw data supporting the conclusions of this article will be made available by the authors, without undue reservation.

## Ethics statement

Ethical review and approval was not required for the study on human participants in accordance with the local legislation and institutional requirements. The patients/participants provided their written informed consent to participate in this study.

## Author contributions

AB led the workshop, supported by YW and SK, and carried out the initial data analysis. LG, NP, and EW-W coordinated the academic initiative underlying this work. All authors contributed to the writing and revision of the manuscript. All authors contributed to the article and approved the submitted version.

## Funding

AHRC GCRF COVID-19 Urgency fund, ‘Belief in the time of Covid-19: Understanding the making of meaning and trust to maximise public health responsiveness of faith communities in DR Congo’. 10th November 2020 - 9th November 2021. Project RA5203.

## Conflict of interest

The authors declare that the research was conducted in the absence of any commercial or financial relationships that could be construed as a potential conflict of interest.

## Publisher's note

All claims expressed in this article are solely those of the authors and do not necessarily represent those of their affiliated organizations, or those of the publisher, the editors and the reviewers. Any product that may be evaluated in this article, or claim that may be made by its manufacturer, is not guaranteed or endorsed by the publisher.

## Footnotes

^1^The full measures were: Prohibition of all gatherings of more than 20 people in public places outside the family home; Closure of schools, universities, official and private higher institutes throughout the national territory. Suspension of all worship. Suspension of sports activities in stadiums and other sports gathering places until further notice; Prohibition, until further notice, of the opening of nightclubs, bars, cafes, terraces and restaurants; Prohibition of the organization of bereavement in rooms and homes. The body will be taken directly from the morgue to the place of burial with a limited number of accompanying persons.
